# Burden of Obesity in Adults at a Tertiary Care Hospital

**DOI:** 10.7759/cureus.84776

**Published:** 2025-05-25

**Authors:** John Bukasa Kakamba, Shruti Wadhwani, Esperant Ntambue Malu, Nikita Wadhwani, Françoise Latil-Plat, Nickson Poka, Isis Kapinga Kalambayi, Manasse Bukasa Mutombo, Abdifitah Mohamed, Ayrton I Bangolo, Jean René M’buyamba

**Affiliations:** 1 Endocrinology, Metabolism, and Nuclear Medicine, Kinshasa University Clinics, Kinshasa, COD; 2 Endocrinology, Liège University Hospital Center, Liège, BEL; 3 Endocrinology and Diabetes, Avignon Hospital Center, Avignon, FRA; 4 Internal Medicine, Hackensack Meridian Health, Palisades Medical Center, North Bergen, USA; 5 Public Health, Kasai Central Provincial Health Division, Ministry of Health, Kinshasa, COD; 6 Diabetes and Endocrinology, National Diabetes Control Program, Ministry of Health, Kinshasa, COD; 7 Ophthalmology, Avignon Hospital Center, Avignon, FRA; 8 Internal Medicine, University of Washington, Seattle, USA; 9 Hematology and Oncology, John Theurer Cancer Center, Hackensack University Medical Center, Hackensack, USA; 10 Cardiology, Kinshasa University Clinics, Kinshasa, COD

**Keywords:** diabetes mellitus, metabolic dysfunction-associated steatohepatitis (mash), obesity epidemic, obesity epidemiology, obstructive sleep apnea

## Abstract

Background: Obesity is a well-established risk factor for many diseases, including hypertension, diabetes mellitus (DM), cardiovascular disease, and obstructive sleep apnea (OSA). Understanding the prevalence, demographics, and associated complications of obesity at the institutional level is crucial for tailoring effective preventive measures and treatment strategies.

Objective: This study aims to investigate the epidemiology and complications of obesity among patients at the Avignon Hospital.

Methods: This is a retrospective study of patients who consulted the Endocrinology Department at the Avignon Hospital for various endocrine disorders between January 2024 and April 2024. The sociodemographic, clinical, and biochemical variables were collected from both the obese and non-obese cohorts. The chi-square test was used to assess statistically significant associations between obesity and other covariates. Logistic regression analysis was used to determine the risk factors for ischemic heart disease in obese patients.

Results: Among the 240 participants in the study, 54.2% were obese. Obesity was more prevalent among females (57.5%) when compared to males (50.4%), and in individuals aged greater than 60 years (68.8%) as compared to other age groups. Among obese patients, 39.2% had type 1, and 32.3% had type 2 diabetes. Obesity was prevalent in 76.4% of all patients with type 2 diabetes. Hypertension and coronary artery disease were present in 52.3% and 58.4% of all obese patients, respectively. OSA was found in 57.7% of obese patients. Among patients with mild, moderate, and severe sleep apnea, 79.5%, 82%, and 85% were obese, respectively. All patients (n = 16) with a fibrosis-4 index (FIB-4) score > 2.67 and 62% of patients with an indeterminate score between 1.3 and 2.67 were obese. Obesity was noted among 83.3% of chronic kidney disease patients and 66.7% of patients with hypertriglyceridemia. Logistic regression analyses revealed that DM and older age were predictive of ischemic heart disease in obese patients.

Conclusion: The majority of our study cohort consisted of obese patients. Obesity was highly prevalent among patients with DM, OSA, metabolic steatohepatitis-related fibrosis, and chronic kidney disease. Older age, DM, chronic kidney disease, and OSA were associated with a greater risk for ischemic heart disease in obese patients.

## Introduction

Non-communicable diseases, including diabetes mellitus (DM), hypertension (HTN), dyslipidemia, and obesity, are significant public health problems due to their widespread prevalence and associated morbidity. These diseases are responsible for 41 million deaths per year, or 74% of all deaths worldwide. About 17 million people die from a non-communicable disease every year before reaching the age of 70 years. A total of 86% of these premature deaths occur in low to middle-income countries [1]. Obesity, defined as excessive and/or abnormal accumulation of adipose tissue in the body, is deemed to be a mechanistic factor in the causation and progression of DM, HTN, obstructive sleep apnea (OSA), metabolic-dysfunction associated steatohepatitis (MASH), dyslipidemia, and osteoarthritis. It is the second leading cause of preventable deaths, following smoking [2]. The prevalence of obesity is increasing at an alarming rate, according to the World Health Organization (WHO), with one in eight people living with obesity in the year 2022. The adult and adolescent obesity estimates have more than doubled and quadrupled, respectively. In 2022, 2.5 billion adults (43%) were overweight, and of these, 890 million (16%) were obese. A total of 37 million children under the age of five years, and over 390 million adolescents and children aged five years and above, were overweight in 2022. Of these, 160 million met the criteria for obesity [3]. Obesity is indeed a pandemic that has impacted even resource-scarce countries. Treatment efforts targeting obesity can help lower associated cardiometabolic risks and ameliorate the public health burden of a plethora of obesity-driven diseases. Obesity is not merely a problem of personal responsibility, rather, it is a complex disease of multifactorial etiology that gives rise to a chronic inflammatory state. The implicit stigma accompanying it often prevents patients from seeking medical care. However, the recent increase in research funding to assess the efficacy of novel anti-obesity therapeutics has destigmatized this condition and paved the way for anti-obesity research.

Weight loss of 5% to 10% can significantly improve the health and quality of life of obese patients, and eventually lessen the economic burden on the public health infrastructure [4]. Given the multiplicity of cultures, ethnicities, and lifestyle choices, it is important to strategically devise plans that are logistically feasible to implement in a given geographical area. The endocrinology department at our hospital has witnessed a notable rise in obesity consultations. Reliable estimates about the prevalence of obesity in the city of Avignon do not exist in the literature. We hope to ascertain the local epidemiology of obesity in Avignon by studying a representative subpopulation of patients at the Avignon Hospital and subsequently implement targeted initiatives to tackle obesity. This study will allow us to identify the extent of obesity and associated comorbidities among the population of Avignon and propose practical solutions from a public health standpoint.

Objectives

Our primary objective was to describe the local burden of obesity and its complications within our study cohort. The secondary objective was to identify the risk factors for ischemic heart disease in obese patients.

## Materials and methods

Study population

This is a retrospective study of patients who consulted the Endocrinology Department at the Avignon Hospital Center (AHC) between January and April 2024 for various endocrine disorders. This hospital serves as a premier tertiary care center, providing premier subspecialty care to more than half a million people residing within Avignon and several adjacent cities. The patients in this study were referred to the endocrinology department either by their primary care physicians or through hospital-based referral programs for evaluation of various endocrine-related conditions. Data on 240 eligible adult patients were collected, of whom 130 were classified as obese. Relevant study variables were obtained from a retrospective chart review. A total of 32 patient records with missing information were excluded from the analysis.

Study variables

Socio-demographic variables included age, gender, and alcohol and/or nicotine consumption. Clinical parameters included body mass index (BMI), obesity, DM, HTN, coronary artery disease (CAD), MASH, orthopedic disorders, OSA, vascular disease, physical activity level, and relevant family history. Biochemical assessments included creatinine clearance, lipid profile, serum albumin, thyroid-stimulating hormone (TSH), vitamin B12, vitamin D, folate, and glycated hemoglobin (HbA1c) levels.

Definitions

The Quetelet index was used to define obesity in this study. Obesity was categorized into three grades: grade 1 (BMI = 30.0-34.9 kg/m²), grade 2 (BMI = 35.0-39.9 kg/m²), and grade 3 (BMI ≥ 40.0 kg/m²). Hypertension was defined as a systolic blood pressure ≥ 140 mmHg or a diastolic blood pressure ≥ 90 mmHg. Cardiovascular risk factors included age, gender, substance use disorder, HTN, DM, chronic kidney disease (CKD), OSA, obesity, hypertriglyceridemia, and physical inactivity. Diabetes mellitus was defined based on a prior diagnosis, the use of antidiabetic medication, a fasting blood glucose level ≥ 126 mg/dL, or an HbA1c level ≥ 6.5%. Diabetic retinopathy was classified through indirect ophthalmoscopic examination as non-proliferative (mild, moderate, or severe) or proliferative. OSA was confirmed by polysomnography and categorized as mild (apnea-hypopnea index (AHI) = 5-14 events/hour), moderate (AHI = 15-29 events/hour), or severe (AHI ≥ 30 events/hour). Renal insufficiency was defined as a creatinine clearance (CrCl) < 60 mL/min. Hypertriglyceridemia was indicated by triglyceride levels > 200 mg/dL. Vitamin D deficiency was defined as a serum vitamin D level < 20 ng/mL, vitamin B12 deficiency as a level < 200 pg/mL, and folic acid deficiency as a serum folate level < 2 ng/mL.

Institutional review board approval

The study protocol was reviewed and granted approval by the Institutional Review Board at the Avignon Hospital. Written informed consent was obtained from all study participants prior to data collection, curation, and analysis. Participants’ data were anonymized to protect their privacy.

Statistical analysis

Collected data were entered into EpiData 3.1 (EpiData Association, Odense, Denmark) and analyzed using SPSS software version 25 (IBM Corp., Armonk, NY). Categorical variables were presented as counts and percentages to summarize their distribution among obese and non-obese patients. Quantitative variables were summarized using measures of central tendency and dispersion. Means and standard deviations were reported for normally distributed variables, while medians and interquartile ranges were used for non-normally distributed variables. The chi-square test was used to assess statistically significant associations between obesity and other covariates. Multiple regression analysis was employed to assess the strength and independence of the associations between ischemic heart disease and presumed risk factors among obese patients. A p-value ≤ 0.05 was considered statistically significant.

## Results

Of the 240 participants in our study cohort, 130 (54.2%) were obese. Table 1 indicates the demographic profile of study participants. Obese patients had a median age of 53 years (interquartile range (IQR): 36-67 years) as compared to 47 years in non-obese subjects (IQR: 33-58). The prevalence of obesity was highest among the elderly (n = 54; 68.8%), followed by patients in the third (n = 21; 52.5%) and fourth (n = 24; 52.2%) decades of their lives, respectively. Obesity was more prevalent among female participants (n = 73; 57.5%) as opposed to males (n = 57; 50.4%). Among the total population, non-Hispanic White individuals comprised the majority of the cohort, with 116 participants, of whom 65 (56%) were obese and 51 (44%) were not. The non-Hispanic Black group consisted of 40 participants, of whom 24 (60%) were obese and 16 (40%) were not. The Hispanic population comprised 84 participants, with a nearly equal distribution of 41 (49%) in the obese category and 43 (51%) in the non-obese category. Among the 65 patients earning less than $50,000 annually, 26 (40%) were obese, while 39 (60%) were not. In the $50,000-$75,000 income bracket, there were 108 participants, with 64 (59.3%) classified as obese and 44 (40.7%) as not obese. Finally, for individuals earning over $75,000, 67 participants were recorded, of whom 40 (59.7%) had obesity and 27 (40.3%) did not.

**Table 1:** Socio-demographic profile of study participants. P ≤ 0.05 was considered statistically significant.

Features	Obese (130), n (%)	Non-obese (110), n (%)	Total, n (%)	p
Median age (interquartile range)	53 (67-36)	47 (58-33)	49 (63-35)	0.63
Age group (years)				0.03
<20	4 (40)	6 (60)	10 (100)	
20-29	12 (46.2)	14 (53.8)	26 (100)	
30-39	21 (52.5)	19 (47.5)	40 (100)	
40-49	24 (52.2)	22 (47.8)	46 (100)	
50-59	16 (40)	24 (60)	40 (100)	
≥60	53 (68.8)	25 (31.2)	77 (100)	
Sex				0.30
Female	73 (57.5)	54 (42.5)	127 (100)	
Male	57 (50.4)	56 (49.6)	113 (100)	
Race				
Non-Hispanic White	65 (56)	51 (44)	116 (100)	0.42
Non-Hispanic Black	24 (60)	16 (40)	40 (100)	
Hispanic	41 (49)	43 (51)	84 (100)	
Annual income				
Less than $50,000	26 (40)	39 (60)	65 (100)	0.27
$50,000-$75,000	64 (59.3)	44 (40.7)	108 (100)	
>$75,000	40 (59.7)	27 (40.3)	67 (100)	
Marital status				0.66
Single	13 (59.1)	9 (40.9)	22 (100)	
Married	117 (53.7)	101 (46.3)	218 (100)	

Nicotine and alcohol use disorders were present in 47 (36.15%) and 14 (10.76%) obese patients, respectively; 43 obese patients (33%) did not have any substance use disorder, whereas the remaining 26 (22%) were using both nicotine and alcohol simultaneously (Table 2). DM was present in 93 obese patients (71.5%); 51 patients (39.2%) had type 1 DM, whereas 42 (32.3%) had type 2 DM. The prevalence of diabetes among non-obese participants was 79%. Among non-obese patients (n = 110), 74 (67.3%) had type 1 DM, whereas 13 (11.8%) had type 2 DM. Obesity was prevalent in 76.4% (n = 42) of all patients with type 2 DM (n = 55).

**Table 2:** Clinical characteristics of the study participants. P ≤ 0.05 was considered statistically significant. CV: cardiovascular; DM: diabetes mellitus; CVA: cerebrovascular accident; CAD: coronary artery disease; HTN: hypertension; PCOS: polycystic ovarian syndrome; PAD: peripheral artery disease; FIB-4: fibrosis-4 index.

Variable	Obese, n (%)	Non-obese, n (%)	Total, n (%)	p
Substance use disorder				0.81
None	43 (57.3)	32 (42.7)	75 (100)	
Alcohol	14 (48.3)	15 (51.7)	29 (100)	
Nicotine	47 (55.3)	38 (44.7)	85 (100)	
Both	26 (51.0)	25 (49.0)	51 (100)	
Diabetes mellitus				<0.001
Absent	37 (61.7)	23 (38.3)	60 (100)	
Type 1	51 (40.8)	74 (59.2)	125 (100)	
Type 2	42 (76.4)	13 (23.6)	55 (100)	
CV risk factors				0.001
None	22 (51.2)	21 (48.8)	43 (100)	
One	4 (25.0)	12 (75.0)	16 (100)	
Two	10 (34.5)	19 (65.5)	29 (100)	
Three	22 (46.8)	25 (53.2)	47 (100)	
Four	70 (68.0)	33 (32.0)	103 (100)	
Five	2 (100)	0 (0.0)	2 (100)	
Family history				0.105
DM 1	8 (40.0)	12 (60.0)	20 (100)	
DM 2	53 (59.6)	36 (40.4)	89 (100)	
CVA	10 (62.5)	6 (37.5)	16 (100)	
CAD	6 (66.7)	3 (33.3)	9 (100)	
HTN	15 (40.5)	22 (59.5)	37 (100)	
PCOS	1 (14.3)	6 (85.7)	7 (100)	
HTN + DM2	16 (72.7)	6 (27.3)	22 (100)	
CVA + DM2	7 (43.8)	9 (56.3)	16 (100)	
Cancer	3 (42.9)	4 (57.1)	7 (100)	
Physical activity per week				0.450
None	61 (55.0)	50 (45.0)	111 (100)	
Once	2 (33.3)	4 (66.7)	6 (100)	
2 days	17 (44.7)	21 (55.3)	38 (100)	
3 days	32 (64.0)	18 (36.0)	50 (100)	
4 days	11 (55.0)	9 (45.0)	20 (100)	
5 days	7 (46.7)	8 (53.3)	15 (100)	
CAD				0.406
Absent	85 (52.1)	78 (47.9)	163 (100)	
Present	45 (58.4)	32 (41.6)	77 (100)	
HTN				0.303
Yes	68 (57.6)	50 (42.4)	118 (100)	
No	62 (50.8)	60 (49.2)	122 (100)	
Retinal complications				0.623
None	44 (56.4)	34 (43.6)	78 (100)	
Minimal nonproliferative retinopathy	16 (44.4)	20 (55.6)	36 (100)	
Moderate nonproliferative retinopathy	30 (50.8)	29 (49.2)	59 (100)	
Severe nonproliferative retinopathy	27 (60.0)	18 (40.0)	45 (100)	
Proliferative retinopathy	13 (59.1)	9 (40.9)	22 (100)	
Hepatic steatosis/fibrosis				<0.001
FIB-4 score < 1.3	93 (48.9)	97 (51.1)	190 (100)	
1.3-2.67	21 (61.8)	13 (38.2)	34 (100)	
>2.67	16 (100)	0 (0.0)	16 (100)	
Diabetic nephropathy				<0.001
Present	41 (28.3)	104 (71.7)	95 (100)	
Absent	89 (93.7)	6 (6.3)	144 (100)	
Joint complications				<0.001
None	82 (44.6)	102 (55.4)	184 (100)	
Carpal tunnel	5 (62.5)	3 (37.5)	8 (100)	
Herniated disc	11 (91.7)	1 (8.3)	12 (100)	
Osteoarthritis	26 (89.7)	3 (10.3)	29 (100)	
Carpal tunnel + herniated disc	6 (85.7)	1 (14.3)	7 (100)	
Vascular complications				0.284
None	82 (52.6)	74 (47.4)	156 (100)	
PAD	6 (75.0)	2 (25.0)	8 (100)	
CVA	3 (100)	0 (00.0)	3 (100)	
CAD	36 (55.4)	29 (44.6)	65 (100)	
Obstructive sleep apnea				<0.001
Absent	55 (37.2)	93 (62.8)	148 (100)	
Light	35 (79.5)	9 (20.5)	44 (100)	
Moderate	23 (82.1)	5 (17.9)	28 (100)	
Severe	17 (85.0)	3 (15.0)	20 (100)	
Parity				0.13
Nullipara	19 (57.6)	14 (42.4)	33 (100)	
Paucipara	44 (56.4)	34 (43.6)	78 (100)	
Multipara	10 (62.5)	6 (37.5)	16 (100)	

More than half of all obese patients (55.4%; n = 72) had at least four cardiovascular risk factors, and 61 patients (47%) had a family history of DM. Hypertension was present in 68 (52.3%) obese patients and 50 (45.5%) non-obese individuals. The proportions of obese (46.9%) and non-obese (45.5%) subjects who were not engaging in physical activity were comparable (61 obese vs. 50 non-obese patients). The prevalence of obesity among patients with severe non-proliferative diabetic retinopathy (DR) (n = 27) and proliferative DR (n = 13) was almost 60% each, but it failed to reach the level of statistical significance. Severe non-proliferative DR was noted in 27 (24.5%) obese patients and 18 (16.4%) non-obese individuals. There was no significant difference in the prevalence of proliferative DR among obese (10%; n = 13) and non-obese (8.2%; n = 9) individuals (p = 0.6). Among patients with CAD, 45 (58.4%) were obese. CAD was prevalent in 34.6% (n = 45) of all obese patients, compared to 29% (n = 32) of non-obese subjects (p = 0.4). Interestingly, all patients (n = 16) with a fibrosis-4 index (FIB-4) > 2.67 were obese. Around 62% (n = 21) of patients with an indeterminate score (between 1.3 and 2.67) were obese (p < 0.001). About 72% of patients (n = 104) with diabetic nephropathy were non-obese (p < 0.001). Orthopedic complications, namely, carpal tunnel syndrome, disc herniation, and osteoarthritis, were more prevalent among obese individuals (p < 0.001). OSA was present in 57.7% (n = 75) of obese patients and 15.5% (n = 17) of non-obese individuals. Among patients with mild, moderate, and severe OSA, 35 (79.5%), 23 (82%), and 17 (85%) patients were obese, respectively.

An elevated HbA1c was noted among 93.8% (n = 122) of all obese patients (Table 3). Among all patients with an elevated HbA1c, 56.7% (n = 122) were obese and 43.3% (n = 93) were not obese (p = 0.02). Furthermore, 83.3% (n = 15) of patients with a lower creatinine clearance (<60 ml/min) were obese (p = 0.01). Two-thirds of all patients (n = 42) with hypertriglyceridemia were obese (p = 0.006). Among subjects with low high-density lipoprotein (HDL) and high low-density lipoprotein (LDL) cholesterol levels, 57.2% (n = 87) and 50.4% (n = 63), respectively, were obese. Of the patients with hypoalbuminemia, 56.4% (n = 92) were obese. Obesity was also prevalent among 65.5% of patients (n = 57) with severe B12 deficiency (p = 0.01). Similarly, 56.8% (n = 63) of patients with folate deficiency were obese. A total of 55% of patients (n = 113) with vitamin D deficiency and 60.4% of patients (n = 29) with hypothyroidism were obese. About one-third of all participants were receiving either statin (n = 81) or anti-hypertensive (n = 72) therapy. Among patients with diabetes, 169 patients (70.4%) were taking insulin, whereas only seven (2.9%) were on a glucagon-like peptide-1 (GLP-1) agonist (Table 4).

**Table 3:** Biochemical profile of the study participants. HbA1c: glycated hemoglobin; CKD: chronic kidney disease; HDL: high-density lipoprotein (mg/dL); LDL: low-density lipoprotein (mg/dL); eGFR: estimated glomerular filtration rate (ml/min/1.73 m^2^); TG: triglycerides (mg/dL). Serum albumin levels measured are in g/dL. P ≤ 0.05 was considered statistically significant.

Variable	Obese, n (%)	Non-obese, n (%)	Total, n (%)	p
HbA1c				0.021
≤6.5%	8 (32.0)	17 (68.0)	25 (100)	
>6.5%	122 (56.7)	93 (43.3)	215 (100)	
CKD				0.012
eGFR < 60	15 (83.3)	3 (16.7)	18 (100)	
eGFR ≥ 60	115 (51.8)	107 (48.2)	222 (100)	
TG				0.006
Normal	88 (49.7)	89 (50.3)	177 (100)	
High	42 (66.7)	21 (33.3)	63 (100)	
HDL cholesterol				0.228
Normal	43 (48.9)	45 (51.1)	88 (100)	
Low	87 (57.2)	65 (42.8)	152 (100)	
LDL cholesterol				0.245
Normal	67 (58.3)	48 (41.7)	115 (100)	
High	63 (50.4)	62 (49.6)	125 (100)	
Serum albumin				0.501
Normal	38 (49.3)	39 (50.7)	77 (100)	
Low	92 (56.4)	71 (46.3)	163 (100)	
Vitamin B12 deficiency				0.013
Absent	77 (49)	80 (51)	157 (100)	
Present	57 (65.5)	30 (34.5)	87 (100)	
Folic acid deficiency				0.294
Absent	67 (51.9)	62 (48.1)	129 (100)	
Present	63 (56.8)	48 (43.2)	111 (100)	
Vitamin D deficiency				0.472
Absent	17 (48.6)	18 (51.4)	35 (100)	
Present	113 (55.1)	92 (44.9)	205 (100)	
Thyroid function				0.304
Euthyroid	92 (51.7)	86 (48.3)	178 (100)	
Hyperthyroid	9 (64.3)	5 (35.7)	14 (100)	
Hypothyroid	29 (60.4)	19 (39.6)	48 (100)	

**Table 4:** Therapeutic profile of the study participants. GLP-1A: glucagon-like peptide 1 agonist.

Therapy	Number of participants (%)	
GLP-1A		
Yes	7 (2.9)	
No	233 (97.1)	
Statins		
Yes	81 (33.7)	
No	159 (66.3)	
Insulin		
Yes	169 (70.4)	
No	71 (29.6)	
Insulin pump		
Yes	129 (53.7)	
No	111 (46.3)	
Anti-hypertensive		
Yes	72 (30)	
No	168 (70)	

We sought to determine the risk factors for ischemic heart disease (IHD) in obese patients. Multivariate logistic regression analysis revealed that older age, DM, CKD, and OSA were predictive of IHD (Table 5). Older individuals (age > 60 years) were 10 times more likely to have IHD compared to younger individuals, after adjusting for other variables. Diabetes and CKD were each associated with an approximately four-fold increase in the likelihood of IHD, relative to individuals without these conditions. In contrast, OSA was linked to a two-fold increase in IHD risk. Gender, substance use, HTN, family history of DM, physical activity, and hypertriglyceridemia were not associated with IHD in our study cohort.

**Table 5:** Univariate and multivariate analyses identifying the risk factors of ischemic heart disease in obese patients. P ≤ 0.05 was considered statistically significant. Significant values have been highlighted with an asterisk (*). CKD: chronic kidney disease; DM: diabetes mellitus; HTN: hypertension; OSA: obstructive sleep apnea.

Variables	OR (95% CI)		p	Adjusted OR (95% CI)		p
Age group (years)						
<20	1		N/A	1		N/A
20-29	1.3 (0.3-5.6)		0.74	1.6 (0.2-9.6)		0.63
30-39	1.6 (0.4-6.8)		0.48	6.6 (0.8-52.4)		0.07
40-49	1.6 (0.4-6.6)		0.49	3.4 (0.4-24.9)		0.23
50-59	0.9 (0.2-3.9)		0.96	2.5 (0.3-20.6)		0.38
≥60	3.3 (0.8-12.8)		0.08	10.0 (1.3-77.1)		0.03*
Male gender	0.7 (0.4-1.2)		0.28	1.1 (0.7-1.5)		0.12
Substance use disorder						
None	1		N/A	1		N/A
Alcohol	0.7 (0.3-1.6)		0.41	0.8 (0.2-1.3)		0.28
Tobacco	0.9 (0.5-1.7)		0.8	1.1 (0.3-1.6)		0.42
Alcohol + tobacco	0.8 (0.4-1.6)		0.48	1.2 (0.5-1.4)		0.35
Physical activity per week						
Five times	1		N/A	1		N/A
Four times	1.4 (0.5-4.1)		0.55	1.1 (0.7-3.1)		0.27
Thrice	0.6 (0.1-4.1)		0.58	0.4 (0.3-2.9)		0.34
Twice	0.9 (0.3-3.1)		0.9	0.6 (0.5-2.4)		0.72
Once	2.0 (0.6-6.5)		0.23	1.3 (0.4-3.8)		0.11
None	1.4 (0.3-9.3)		0.63	0.9 (0.5-5.6)		0.47
HTN	1.3 (0.8-2.2)		0.29	1.5 (0.9-1.9)		0.16
Family history of DM	0.6 (0.4-1.2)		0.18	1.6 (0.6-4.3)		0.32
DM	2.8 (1.2-6.7)		0.02*	3.9 (1.1-12.8)		0.03*
Hypertriglyceridemia	1.8 (1.0-3.4)		0.31	1.8 (0.7-4.6)		0.25
CKD	2.3 (1.3-3.6)		0.04*	3.7 (1.6-5.4)		0.01*
OSA	1.4 (0.8-2.5)		0.37	2.2 (1.3-4.6)		0.02*

## Discussion

The objective of our study was to ascertain the prevalence of obesity and associated complications in the city of Avignon. It is paramount to determine the inciting factors and monitor the prevailing trends of obesity and associated complications in Avignon because these data will help define the magnitude of the obesity epidemic and provide a framework to develop locally actionable plans aimed at combating obesity. We analyzed data from 240 participants who sought obesity consultation at the Avignon Hospital Center. More than half of our study cohort was obese (54.2%; n = 130), and the prevalence of obesity was higher among females (57.5%; n = 73) as compared to males (50.4%; n = 57). These profound statistics are on par with the global obesity trends and likely stem from a combination of factors, namely, poor dietary choices, sedentary lifestyles, suboptimal metabolism, etc. The prevalence of obesity was the highest among elderly patients (68.8%; n = 53), followed by patients aged 30-40 years (52.5%; n = 21) and 40-50 years (52.2%; n = 24), respectively. The prevalence of obesity was marginally higher among females compared to males in our study cohort.

We also explored the relationship between obesity and several other comorbidities. Obesity was found to be significantly associated with type 2 DM, OSA, metabolic dysfunction-associated steatotic liver disease (MASLD), including advanced fibrosis, and orthopedic complications, namely, disc herniation, osteoarthritis, etc., in our study cohort. These findings are consistent with contemporary literature, which incriminates obesity in the etiopathogenesis of the aforementioned complications. Optimal management of obesity is paramount to mitigating and/or resolving these chronic conditions.

The lifetime risk of diabetes increases from ~10% to ~70% if the BMI rises from 18.5 to 35 kg/m^2^ or above [5]. Even modest weight loss (5-10%) and an increase in physical activity (up to 150 min/week) lead to a marked reduction in the incidence of diabetes through enhanced glycemic control. A five-fold reduction in the incidence of diabetes following bariatric procedures has been documented in one study [6]. Weight loss, thus, is a cornerstone of diabetes management.

Recently, a large community-based cohort study analyzed data from 2950 patients with OSA [7]. Approximately 41% of these patients were females with a median age of 65 years and a median BMI of 28.8 kg/m^2^. A higher percentage of females (43.6%) with OSA were obese as compared to males (36.5%). Among patients with OSA, 60.6% were non-obese and 39.4% were obese. Additionally, 54.3% of severe, 42.6% of moderate, and 34.1% of mild OSA patients were obese. Prevalence of obesity was greater among younger adults (<65 years) as compared to older adults (48.3% vs. 30.9%) with OSA. In our study cohort, OSA was present in 57.7% of obese patients and 15.5% of non-obese individuals. Furthermore, 79.5% of mild, 82% of moderate, and 85% of severe OSA patients were obese, respectively.

Obesity plays a key role in the development of osteoarthritis (OA), which is a multifactorial disorder involving abnormal mechanical loading and dysregulated immunoinflammatory responses. In our study, OA was present in 2.7% of non-obese and 20% of obese individuals. In addition to intensive physical therapy, treatment of obesity remains the mainstay of obesity management. A multinational, double-blind, randomized placebo-controlled trial looking at 407 obese patients with moderate to severe OA found significantly greater reductions in body weight and OA-associated pain after weekly semaglutide administration when compared to placebo [8].

MASH is the most common liver disease globally. Its prevalence is rapidly increasing due to an alarming rise in obesity. GLP1 agonists are emerging as potential treatment options for MASH, given the central role of obesity in its pathophysiology. A placebo-controlled trial of semaglutide in patients with MASH showed a statistically significant reduction in MASH than placebo, but the improvement in fibrosis stage was not statistically different between the two groups [9]. Similarly, tirzepatide was more effective than placebo in another randomized controlled trial in terms of MASH resolution without worsening of fibrosis [10]. Recently, another trial assessing the efficacy of dual glucagon and glucagon-like peptide (GLP) agonist, survodutide, interestingly showed improvement in MASH and fibrosis stage in patients with biopsy-confirmed MASH [11]. Only 2.9% (n = 7) of individuals were using a GLP-1A despite the significant prevalence of obesity (54.2%) in our study cohort. It is imperative to institute protocols to eliminate medication access barriers and address logistical challenges that adversely impact the local availability of these therapeutics. Educational interventions that positively affect physicians’ GLP-1A prescription trends and increase medication adherence among patients are also effective ways to adequately leverage the power of these therapeutics.

The prevalence of CKD was significantly greater among obese patients (83.3%; n = 15) as compared to non-obese subjects (16.7%; n = 3). Recent literature has deemed obesity as an independent risk factor for CKD progression. Obesity is associated with several kidney diseases, including focal segmental glomerulosclerosis, nephrolithiasis, IgA nephropathy, and reduced transplant survival rates. It has been postulated that obesity triggers renal hypoxia and hemodynamic disturbances, causing abnormal activation of the renin-angiotensin-aldosterone pathway. The exact mechanisms are unclear and require further elucidation [12].

The majority of patients (65.5%; n = 57) with vitamin B12 deficiency were obese. Several studies have found an association between obesity and vitamin B12 deficiency, but causality has not been definitively established, and further research is warranted. Low vitamin B12 levels impair the generation of methionine from homocysteine, thereby reducing protein synthesis and lean muscle mass. Obesity can be associated with decreased dietary intake, increased catabolism, and/or sequestration of B12 within adipocytes. Alteration of gut microbiome in obese patients can also disrupt B12 metabolism [13].

We sought to determine the risk factors for IHD in obese patients. Binary logistic regression analysis revealed that older age and DM were predictive of IHD (Table 5). Several large prospective studies have indicated that the association between IHD and obesity is largely mediated by DM, HTN, and dyslipidemia [14]. Other prospective analyses, however, have suggested that the risk for CAD in obese patients persists after adjusting for DM, HTN, and dyslipidemia [15,16]. A meta-analysis of 1.8 million people showed that hypertension, elevated cholesterol, and glucose levels explain half of the associations between overweight/obesity and CAD [17]. In our study, older age, DM, CKD, and OSA were independently predictive of IHD in obese individuals. Early institution of upstream interventions aimed at preventing or treating obesity is imperative, as it is an independent predictor of adverse cardiovascular outcomes.

Limitations and future research

Our study has several limitations due to its cross-sectional and descriptive nature. Although we found significant associations between obesity and other covariates, we cannot draw conclusions about causality or generalize our findings to a larger population. Moreover, certain variables, such as substance use, physical activity, and medication adherence, may have been subject to bias, as they were based on self-reported data from study participants. Selection bias is difficult to eliminate, as the data were derived from existing records rather than a random sample. There is also the potential for information bias, as the accuracy and completeness of the data depend on how thoroughly it was recorded in the patient charts. Additionally, causal relationships cannot be established due to the observational nature of the design, and the generalizability of the findings may be limited to the specific population or setting from which the data were drawn. Finally, missing or incomplete data led to the exclusion of several patient records, which could have weakened the robustness of the analysis. Future research should longitudinally assess the relationship between obesity and possible complications and elucidate the impact of targeted interventions in mitigating obesity-related outcomes. These metrics will lend crucial information for the development of local public health policies to tackle obesity. Several public health measures can be enforced to mitigate obesity and associated complications. Implementing policy changes to address food deserts, increase access to healthy foods at subsidized rates, and limit the number of fast food outlets near schools and low-income neighborhoods can help reduce food insecurity and encourage healthier eating. Employee-sponsored initiatives and school-based programs are critical in creating environments that promote health and well-being. When implemented effectively, these programs can address obesity, encourage healthier lifestyles, and provide support for individuals needing weight management interventions.

## Conclusions

Our study offers valuable insights into the epidemiology of obesity in Avignon. The increasing public health burden of obesity and associated complications in the city requires the development of comprehensive public health strategies and multidisciplinary involvement. Identification of individual and societal factors contributing to obesity is paramount. Implementation of educational initiatives and elimination of barriers to healthcare access can be instrumental in abating the epidemic of obesity in the city of Avignon.
